# Changes in essential cancer medicines and association with cancer outcomes: an observational study of 158 countries

**DOI:** 10.1186/s12885-024-13247-w

**Published:** 2024-12-18

**Authors:** Moizza Zia Ul Haq, Camila Heredia, Adelaide Buadu, Amal Rizvi, Aine Workentin, Nav Persaud

**Affiliations:** 1 MAP Centre for Urban Health Solutions, Li Ka Shing Knowledge Institute, St. Michael’s Hospital, Unity Health Toronto, 209 Victoria St, Toronto, ON M5B 1T8 Canada; 2 Department of Family and Community Medicine, University of Toronto, Toronto, ON Canada

**Keywords:** Essential medicines, Cancer medicines, Access to medicines

## Abstract

**Background:**

Cancer is a major cause of mortality worldwide, and differences in cancer mortality rates between countries are, in part, due to differences in access to cancer care, including medicines. National essential medicines lists (NEMLs) play a role in prioritization of healthcare expenditure and access to medicines. We examined the association between amenable cancer mortality and listing medicines used in the management of eight cancers (non-melanoma skin, uterine, breast, Hodgkin lymphoma, colon, leukemia, cervical, and testicular) in national essential medicines lists of 158 countries and summarized changes to the inclusion of cancer treatments in NEMLs.

**Methods:**

We conducted a cross-sectional examination of NEMLs for 158 countries, which were obtained in May 2023. We identified medicines used to treat each of the eight cancers and determined the number of medicines listed by NEMLs for each cancer. We conducted multiple linear regressions to examine the association between the number of medicines listed on the NEMLs and cancer mortality.

**Results:**

We found associations between cancer medicine listing and outcomes for six of the eight examined cancers (non-melanoma skin cancer (*p* = 0.001), uterine cancer (*p* = 0.006), breast cancer (*p* = 0.001), Hodgkin lymphoma (*p* = 0.021), colon cancer (*p* = 0.006), and leukemia (*p* = 0.002)), when adjusting for healthcare expenditure and population size.

**Conclusion:**

There was an association between listing cancer medicines on NEMLs and cancer mortality. Further research is required to explore how cancer mortality may be impacted by other cancer interventions, as well as policies to improve equitable access to cancer care.

**Supplementary Information:**

The online version contains supplementary material available at 10.1186/s12885-024-13247-w.

## Background

Cancer is a major cause of mortality globally – in 2020 alone, there were an estimated 19.3 million new cases of cancer and almost 10 million cancer deaths, and cancer incidence and mortality is rapidly increasing [1]. Differences in mortality rates between countries rates are due to differences in risk factors and access to cancer care, including medicines [1].

The World Health Organization (WHO) has developed and updated Model Lists of Essential medicines, consisting of medicines “intended to be available in functioning health systems at all times, in appropriate dosage forms, of assured quality and at prices individuals and health systems can afford”, [2] which includes various cancer medicines. Several countries have developed their own national essential medicines lists (NEMLs), as per recommendations by the WHO to select essential medicines based on "disease burden, public health relevance, clinical effectiveness and safety, and cost-effectiveness” [3, 4]. These lists play a role in public procurement and supply and prioritization of healthcare expenditure [5]. Medicines deemed essential have been found to be more available than non-essential medicines in both the private and public sectors [6]. Furthermore, NEMLs influence essential medicine reimbursement schemes and in some countries may support the provision of essential medicines at no charge [5, 7]. Thus, NEMLs play a role in access to medicines, including cancer medicines, and may consequently impact cancer mortality.

The value of newer cancer treatments has been questioned since they are not always proven to reduce mortality [8–12]. Focusing on established treatments and basic care may be more important globally than expanding access to newer expensive treatments with questionable value [12]. We found only a weak association between the listing of cancer treatments in essential medicines lists in 2017 and relevant cancer outcomes [13].

The purpose of this study was to summarize changes to the inclusion of cancer treatments in NEMLs and to examine the association between amenable cancer mortality, measured by mortality-to-incidence ratios, and listing medicines used in the management of eight cancers in national essential medicines lists (NEMLs) of 158 countries.

## Methods

### Dataset sources

In May 2023, we obtained NEMLs by performing web searches and contacting experts, and extracted all the medicines listed in the NEMLs for each country. We identified 158 NEMLs, which were used to create an updated Global Essential Medicines (GEM) database of 2084 medicines (Persaud et al.: National essential medicine lists changes between 2017 and 2023: a descriptive study, submitted).

We obtained mortality-to-incidence ratios (MIRs) for each country for eight cancers (non-melanoma [NM] skin, uterine, breast, Hodgkin lymphoma, colon, leukemia, cervical, and testicular) from the 2019 Global Burden of Disease Study, which represent mortality amenable to health-care access and quality [14]. The mortality-to-incidence ratio (MIR) is calculated by dividing the mortality rate for a cancer by the incidence rate. Cancer mortality-to-incidence ratios utilize cancer registry data for each country [14, 15]. We chose these eight cancers because they had readily available mortality data [14].

### Data collection

In December 2023, we searched the WHO Publications website (using the topics cancer, breast cancer, and cervical cancer) for international treatment guidelines for cancer management. We identified 2 guidelines for the management of breast cancer: the Global breast cancer initiative implementation framework, [16] and the WHO EMRO Technical Publication series 31-Guidelines for management of breast cancer, [17] and 4 guidelines for the management of cervical cancer: the Strategic framework for the comprehensive prevention and control of cervical cancer in the Western Pacific Region, [18] the Regional implementation framework for elimination of cervical cancer as a public health problem, [19] the Guide to essential practise for comprehensive cervical cancer control, [20] and 'Best buys' and other recommended interventions for the prevention and control of noncommunicable diseases [21]. We recorded the medicines used in the management of breast and cervical cancers from these 6 guidelines.

To obtain medicines for the management the other six cancers which did not have an international treatment guideline and to obtain additional medicines for the management of breast and cervical cancers, we retrieved medicines from the MEDI-HPS (MEDication Indication resource high precision subset) and the updated MEDI-2 HPS database [22, 23]. We searched using ICD-9-CM and ICD-10-CM diagnosis codes defining each of the eight cancers. We also conducted searches of the MIA database, which maps ICD (International Classification of Diseases) codes to ATC (Anatomical Therapeutic Chemical) medicine codes [24]. Finally, we searched the 2023 WHO Model List of Essential Medicines for medicines that were indicated for any of the eight cancers [2]. If a medicine appeared in any of the searches for a cancer guideline, MEDI-HPS, MIA, or 2023 WHO Model List of Essential Medicines, they were included in the initial list of medicines for that cancer. We created a final list of medicines (see Appendix) for each cancer that only included medicines if they were determined to be clinically appropriate in the management of the cancer. If more than 2 medicines from a drug class (based on ATC codes) appeared in the searches, all other drugs from that class were included in the final list if determined to be clinically appropriate as well. Medicines that were excluded were those used as supportive care, such as analgesics and bisphosphonates.

We coded the list of the medicines associated with each of the eight cancers into the GEM database and identified overlaps on these lists and on each country's NEML. We totalled the overlapping medicines to create a coverage score for each country per cancer cause. For instance, if a country listed 8 of the 14 medicines for NM skin cancer, that country received an NM skin cancer coverage score of 8. The coverage score accounted for the number of medicines by name, and did not account for different dosages or formulations. We obtained data for health expenditure from the WHO Global Health Observatory for the year 2021 [25]. Most of the data pertained to the year 2021; records from 2021 records were unavailable for two countries, so information from the nearest available year was accessed [26, 27]. We obtained population size data from the United Nations for the year 2021 [28]. The data are summarized in Table 1.

**Table 1:** Country characteristics and cancer medicine coverage scores

Country	NEML Year	Health spending US$ per capita (2021)	Population size UN (2021)	Medicine coverage score: NM skin	Medicine coverage score: uterine	Medicine coverage score: breast	Medicine coverage score: Hodgkin	Medicine coverage score: colon	Medicine coverage score: leukemia	Medicine coverage score: cervical	Medicine coverage score: testicular
Afghanistan	2015	81	40,099,462	2	1	3	0	1	2	0	1
Albania	2022	465	2,854,710	1	0	9	2	1	11	1	1
Algeria	2023	205	44,177,969	1	3	12	1	0	7	0	1
Angola	2021	64	34,503,774	9	2	14	10	3	20	3	9
Antigua and Barbuda	2022	923	93,220	6	2	17	7	3	14	2	4
Argentina	2021	1045	45,276,780	0	1	1	0	0	0	0	0
Armenia	2021	613	2,790,974	8	2	18	11	4	19	3	8
Australia	2023	7055	25,921,089	12	4	41	15	7	49	5	9
Bahrain	2015	1146	1,463,266	8	2	29	12	5	33	3	10
Bangladesh	2019	58	169,356,251	5	1	11	10	2	14	1	6
Belarus	2021	468	9,578,168	9	3	36	16	7	42	3	10
Benin	2018	35	12,996,895	6	2	21	9	3	18	2	5
Bhutan	2021	120	777,487	3	1	5	2	2	4	1	1
Bolivia (Plurinational State of)	2022	273	12,079,472	10	2	26	11	6	27	4	9
Bosnia and Herzegovina	2019	692	3,270,943	0	0	0	0	0	0	0	0
Botswana	2012	457	2,588,423	4	1	8	7	1	14	0	6
Brazil	2022	761	214,326,223	2	1	8	1	1	10	1	1
Bulgaria	2023	1040	6,885,868	1	3	18	1	2	18	1	1
Burkina Faso	2020	57	22,100,684	9	2	22	11	5	23	4	9
Burundi	2022	24	12,551,213	9	2	18	9	4	13	4	7
Cabo Verde	2018	248	587,925	9	2	18	10	3	17	4	9
Cambodia	2018	122	16,589,024	7	1	12	9	2	14	3	8
Cameroon	2022	64	27,198,628	9	2	29	14	5	29	4	10
Central African Republic	2017	43	5,457,155	5	1	13	10	3	11	1	5
Chad	2022	36	17,179,740	9	2	22	11	5	25	4	9
Chile	2006	1518	19,493,185	7	2	19	14	2	26	3	10
China	2018	671	1,425,893,465	8	2	15	8	4	23	3	7
Colombia	2019	558	51,516,562	9	3	33	14	6	35	3	10
Comoros	2014	99	821,626	5	1	11	13	2	16	1	7
Congo	2019	81	5,835,806	7	1	17	13	4	16	2	6
Cooks Islands	2017	737	17,003	0	1	3	0	0	1	1	1
Costa Rica	2019	949	5,153,957	8	2	24	10	5	26	4	10
Cote D'Ivoire	2020	82	27,478,249	9	3	31	15	6	27	3	10
Croatia	2022	1384	4,060,136	10	5	42	20	8	56	6	10
Cuba	2018	1186	11,256,373	9	3	28	12	6	34	3	10
Czechia	2012	2499	10,510,751	10	5	39	15	8	40	5	8
Democratic People's Republic of Korea	2012	0.5	25,971,909	1	0	5	2	1	4	1	2
Democratic Republic of the Congo	2020	22	95,894,119	5	1	7	5	1	6	1	3
Djibouti	2007	88	1,105,558	0	0	0	0	0	0	0	0
Dominica	2022	482	72,413	6	2	17	7	3	14	2	4
Dominican Republic	2018	417	11,117,874	9	2	20	13	3	18	4	10
Ecuador	2019	494	17,797,737	9	3	33	14	7	36	3	10
Egypt	2018	180	109,262,178	10	2	27	13	7	30	3	10
El Salvador	2020	442	6,314,168	8	2	17	11	4	20	4	9
Equatorial Guinea	2012	256	1,634,466	0	1	1	0	0	0	0	0
Eritrea	2010	25	3,620,312	5	1	7	7	1	12	0	4
Estonia	2012	2095	1,328,701	3	2	12	4	1	20	0	3
Eswatini	2012	280	1,192,271	5	1	7	5	1	5	0	3
Ethiopia	2020	26	120,283,026	9	2	25	14	5	32	3	10
Fiji	2015	250	924,610	8	2	13	9	2	14	2	6
Gabon	2019	234	2,341,179	7	1	16	9	7	16	4	7
Gambia	2019	25	2,639,916	4	1	8	5	1	8	2	2
Georgia	2007	417	3,757,980	5	1	10	11	2	15	1	7
Ghana	2017	100	32,833,031	5	1	10	6	0	12	1	6
Greece	2007	1846	10,445,365	11	6	35	14	7	40	4	8
Grenada	2022	505	124,610	6	2	17	7	3	14	2	4
Guatemala	2021	341	17,608,484	9	3	28	11	7	28	4	8
Guinea	2021	45	13,531,906	7	2	18	9	3	19	3	6
Guinea-Bissau	2020	69	2,060,721	9	2	19	10	3	26	3	8
Guyana	2021	471	804,567	10	2	18	8	4	17	3	6
Haiti	2020	58	11,447,569	10	2	25	14	6	27	4	10
Honduras	2018	254	10,278,346	8	2	23	12	7	21	4	10
Iceland	2022	6716	370,335	2	1	4	3	2	6	1	4
India	2022	74	1,407,563,842	10	2	23	12	5	26	3	10
Indonesia	2021	161	273,753,191	9	2	15	11	2	19	3	9
Iran (Islamic Republic of)	2017	393	87,923,433	10	4	38	21	7	52	4	10
Iraq	2014	249	43,533,593	10	3	28	15	6	36	4	10
Ireland	2023	6764	4,986,526	5	4	28	5	3	31	1	3
Jamaica	2015	372	2,827,695	11	2	23	14	4	24	3	10
Japan	2018	4347	124,612,531	0	0	1	2	0	4	0	0
Jordan	2021	299	11,148,278	10	2	25	14	5	32	3	10
Kazakhstan	2020	403	19,196,466	0	0	0	0	1	0	0	1
Kenya	2019	95	53,005,614	10	3	28	15	6	30	6	10
Kiribati	2009	262	128,874	3	1	5	2	1	3	0	2
Kyrgyzstan	2009	73	6,527,744	5	1	15	7	3	13	1	5
Latvia	2023	1898	1,873,919	2	1	22	7	4	28	1	3
Lebanon	2018	307	5,592,631	7	3	24	11	6	28	4	8
Lesotho	2005	115	2,281,455	4	0	4	3	2	5	0	3
Liberia	2022	112	5,193,416	2	1	6	4	1	4	1	3
Libya	2019	381	6,735,277	9	2	29	15	7	31	4	10
Lithuania	2012	1859	2,786,651	8	3	27	12	3	24	4	9
Madagascar	2019	18	28,915,653	8	2	22	13	5	22	4	10
Malawi	2015	47	19,889,742	8	2	15	12	2	15	3	9
Malaysia	2023	487	33,573,874	9	2	27	15	5	35	4	10
Maldives	2021	1039	521,458	10	5	37	15	7	32	6	9
Mali	2019	40	21,904,983	7	1	15	13	4	18	3	8
Malta	2022	3642	526,748	10	2	34	15	7	41	4	10
Marshall Islands	2007	767	42,050	0	1	2	0	0	0	0	0
Mauritania	2021	89	4,614,974	7	2	14	7	3	16	3	5
Mauritius	2022	565	1,298,915	7	1	22	12	4	22	3	10
Mexico	2017	611	126,705,138	10	5	44	17	8	44	4	10
Mongolia	2020	316	3,347,783	7	3	20	6	7	19	4	6
Montenegro	2020	985	627,859	10	2	36	14	7	40	5	9
Morocco	2017	221	37,076,585	9	2	22	11	8	25	3	9
Mozambique	2017	45	32,077,072	9	2	15	9	3	21	4	9
Myanmar	2016	65	53,798,085	10	2	29	14	6	34	4	10
Namibia	2016	456	2,530,151	8	1	16	12	3	22	2	9
Nauru	2010	1530	12,512	0	1	3	0	0	1	0	1
Nepal	2021	65	30,034,990	10	2	21	13	3	24	4	10
Nicaragua	2011	198	6,850,540	7	1	10	8	2	16	2	9
Niger	2018	34	25,252,722	3	1	9	5	3	7	2	3
Nigeria	2020	84	213,401,323	10	2	28	13	5	30	4	10
Niue	2006	1912	1,937	0	1	2	0	0	0	0	0
North Macedonia	2015	560	2,103,330	9	1	12	11	2	18	3	10
Oman	2020	853	4,520,471	5	4	33	10	6	40	5	5
Pakistan	2021	43	231,402,117	10	2	27	14	6	34	5	10
Palau	2017	2045	18,024	1	1	3	0	0	2	1	1
Panama	2019	1415	4,351,267	2	0	7	3	0	9	0	1
Paraguay	2009	479	6,703,799	8	2	13	8	3	12	3	8
Peru	2018	412	33,715,472	9	2	29	14	7	33	4	10
Philippines	2022	203	113,880,328	9	3	29	11	5	27	4	10
Poland	2017	1159	38,307,726	9	5	39	15	7	50	5	9
Portugal	2020	2747	10,290,103	0	0	0	0	0	0	0	0
Republic of Korea	2019	3260	51,830,139	2	1	7	8	2	11	2	3
Republic of Moldova	2021	410	3,061,507	10	2	30	14	6	38	4	10
Romania	2021	963	19,328,560	8	2	23	13	6	27	3	10
Russian Federation	2019	936	145,102,755	10	3	40	18	9	46	4	9
Rwanda	2022	60	13,461,888	9	2	24	10	4	26	4	8
Saint Kitts and Nevis	2022	1114	47,607	6	2	17	7	3	14	2	4
Saint Lucia	2022	585	179,652	6	2	17	7	3	14	2	4
Saint Vincent and Grenadines	2022	448	104,332	6	2	17	7	3	14	2	4
Sao Tome and Principe	2022	186	223,108	5	1	12	8	2	13	1	5
Saudi Arabia	2020	1442	35,950,396	10	2	29	16	6	33	5	10
Senegal	2018	71	16,876,720	7	2	22	9	4	15	3	7
Serbia	2022	919	7,296,769	9	4	40	19	7	43	5	10
Seychelles	2022	718	106,471	7	1	11	5	4	8	3	4
Sierra Leone	2021	43	8,420,641	5	1	12	6	2	8	2	3
Slovakia	2023	1685	5,447,622	9	4	37	10	7	38	5	5
Slovenia	2017–2023	2775	2,119,410	11	5	44	19	9	56	6	10
Solomon Islands	2017	106	707,851	3	1	9	6	2	12	2	4
Somalia	2019	33	17,065,581	9	2	23	13	5	27	4	10
South Africa	2020–2021	584	59,392,255	9	2	30	12	6	30	4	9
South Sudan	2018	33	10,748,273	10	2	21	12	5	23	4	10
Spain	2019	3234	47,486,935	0	0	0	0	0	1	0	0
Sri Lanka	2019	166	21,773,441	0	1	1	0	0	0	0	0
Sudan	2014	22	45,657,202	9	3	22	12	3	23	3	10
Suriname	2022	299	612,985	8	2	15	12	4	17	3	6
Sweden	2023	6901	10,467,097	3	1	6	0	1	3	0	1
Syrian Arab Republic	2019	89	21,324,367	9	2	21	12	5	30	3	10
Tajikistan	2009	73	9,750,064	6	1	10	9	1	15	1	9
Thailand	2021	364	71,601,103	9	4	28	14	5	32	5	10
Timor-Leste	2015	135	1,320,942	2	1	3	0	2	4	0	1
Togo	2012	54	8,644,829	7	2	13	7	2	13	2	7
Tonga	2007	279	106,017	1	1	3	0	0	1	0	1
Trinidad & Tobago	2019	1125	1,525,663	11	2	32	14	7	30	4	10
Tunisia	2012	265	12,262,946	5	3	21	8	4	23	4	6
Tuvalu	2010	1071	11,204	1	1	5	1	1	3	0	1
Uganda	2016	43	45,853,778	10	2	21	12	5	22	4	10
Ukraine	2017	368	43,531,422	0	1	5	2	0	5	0	0
United Republic of Tanzania	2021	37	63,588,334	9	2	22	10	5	21	4	10
Uruguay	2020	1620	3,426,260	10	4	38	15	7	41	5	9
Uzbekistan	2021	157	34,081,449	7	2	19	9	7	19	4	7
Vanuatu	2014	133	319,137	0	1	4	1	0	2	0	1
Venezuela (Bolivarian Republic of)	2015	160	28,199,867	9	2	20	12	4	25	4	10
Viet Nam	2018	173	97,468,029	7	1	13	11	2	16	3	10
Yemen	2019	63	32,981,641	10	2	22	12	5	24	2	9
Zambia	2020	75	19,473,125	9	2	16	15	2	22	4	9
Zimbabwe	2020	63	15,993,524	2	1	3	0	0	2	1	1

### Data analysis

To identify changes to the inclusion of cancer treatments in NEMLs, we compared our updated dataset to the 2017 Global Essential Medicines Database [29]. For each cancer medicine, we tabulated the number of countries that listed that medicine in our updated dataset but did not list that medicine in the 2017, and the number of countries that listed that medicine in the 2017 dataset but did not list it in our updated dataset. For our second aim, we conducted our analysis using Stata (16, StataCorp LLC, College Station, TX) for each model, and used multiple linear regression to assess the association between the MIRs and the number of medicines listed in each country's NEML for each cancer (based on the coverage scores). Statistical significance was set at a *p*-value ≤ 0.05. The co-efficient for each medicine and co-variate, the lower 95% CI (confidence interval), the upper 95% CI, and *p*-value of the association were recorded. We included healthcare expenditure and population size as they are generally known to influence health systems. The regression was run both unadjusted and adjusted with healthcare expenditure and population. We also ran a model using linear regression to assess the association between MIR and healthcare expenditure for each of the eight cancers.

### Role of the funding source

Our funding sources had no role in the study design; in the collection, analysis, and interpretation of data; in the writing of the report; and in the decision to submit the paper for publication.

## Results

We collected healthcare expenditure per capita, and population data for all 158 countries with NEMLs for each of the eight cancer causes (see Table 1).

Across the 158 NEMLs, a median of 33 (IQR: 29.5 (19.25–48.75), range: 0–98) cancer treatments were included. The most commonly listed medicines were methotrexate (listed by 145 countries, 92%), medroxyprogesterone (listed by 137 countries, 87%), tamoxifen (listed by 135 countries, 85%), and cyclophosphamide (listed by 131 countries, 83%). Compared with NEMLs from 2017, the most commonly added medicines were docetaxel (added by 46 countries), anastrozole (added by 44 countries), capecitabine (added by 44 countries), oxaliplatin (added by 43 countries), and paclitaxel (added by 40 countries). We also identified 29 cancer medicines that were listed by at least one country in our updated database that were not on any NEML from the 2017 database analysis. These included pembrolizumab (on 17 NEMLs), ibrutinib (on 15 NEMLs), and ruxolitinib (on 15 NEMLs). Of these 29 new medicines, most were not on the WHO Model Lists, except for prembrolizumab, which was added to the 2019 Model List, and ibrutinib, which was added to the 2021 Model List.

We identified 14 medicines for NM skin cancer, 6 for uterine cancer, 55 for breast cancer, 26 for Hodgkin lymphoma, 10 for colon cancer, 73 for leukemia, 6 for cervical cancer, and 10 for testicular cancer. The median medicine coverage scores and IQR for each cancer were: NM skin cancer 8 (IQR 4.75 (4.25–9)), uterine cancer 2 (IQR 1 (1–2)), breast cancer 18 (IQR 17 (10–27)), Hodgkin lymphoma 10 (IQR 7.75 (5.25–13)), colon cancer 3 (IQR 4 (2–6)), leukemia 19 (IQR 17.5 (11.25–28.75), cervical cancer 3 (IQR 3 (1–4), and testicular cancer 8 (IQR 7 (3–10)).

**Fig. 1:**
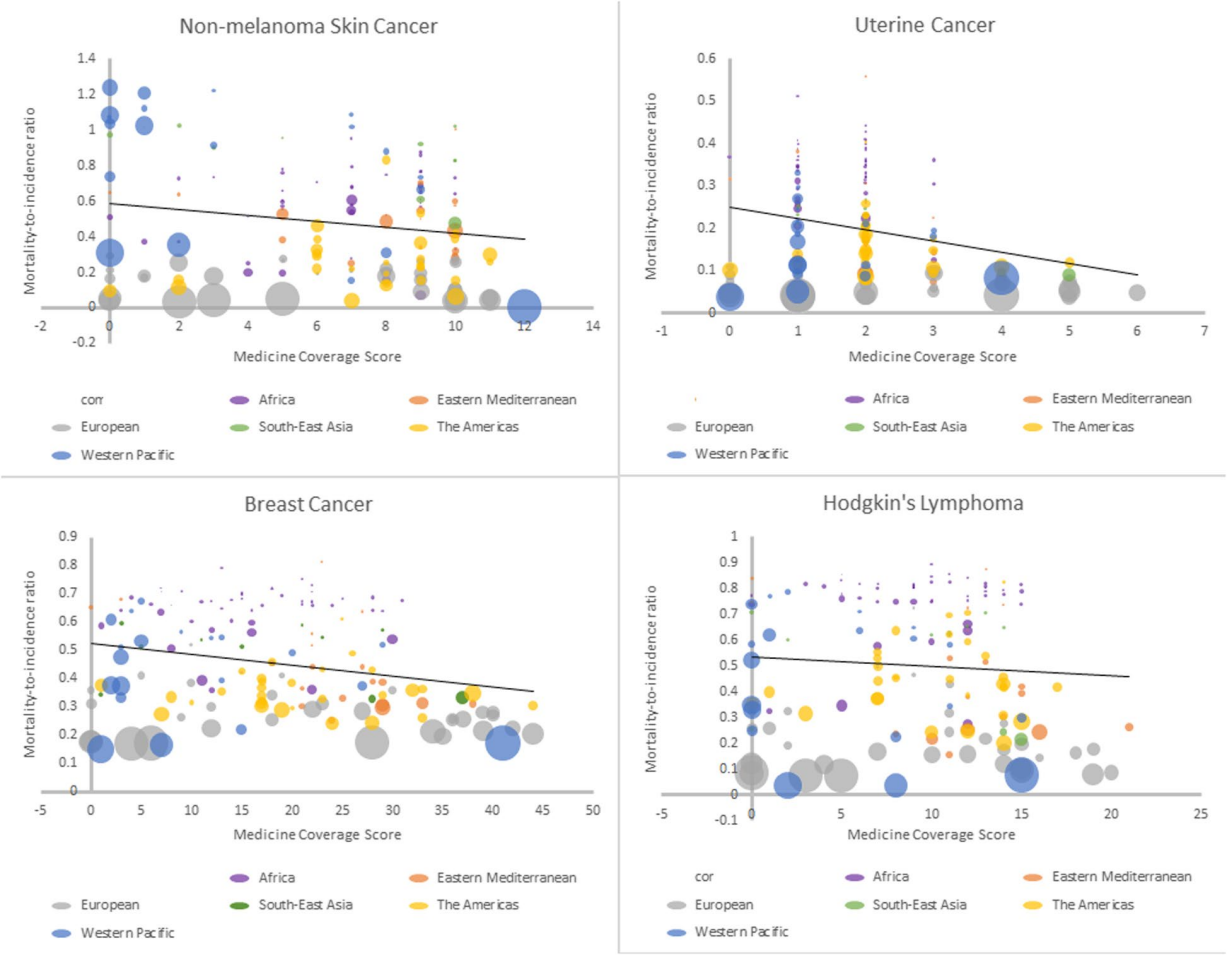
Relationship between medicine coverage score and mortality-to-incidence ratio for NM skin cancer, uterine cancer, breast cancer, and Hodgkin’s lymphoma, with the size of the bubbles representing healthcare expenditure

**Fig. 2:**
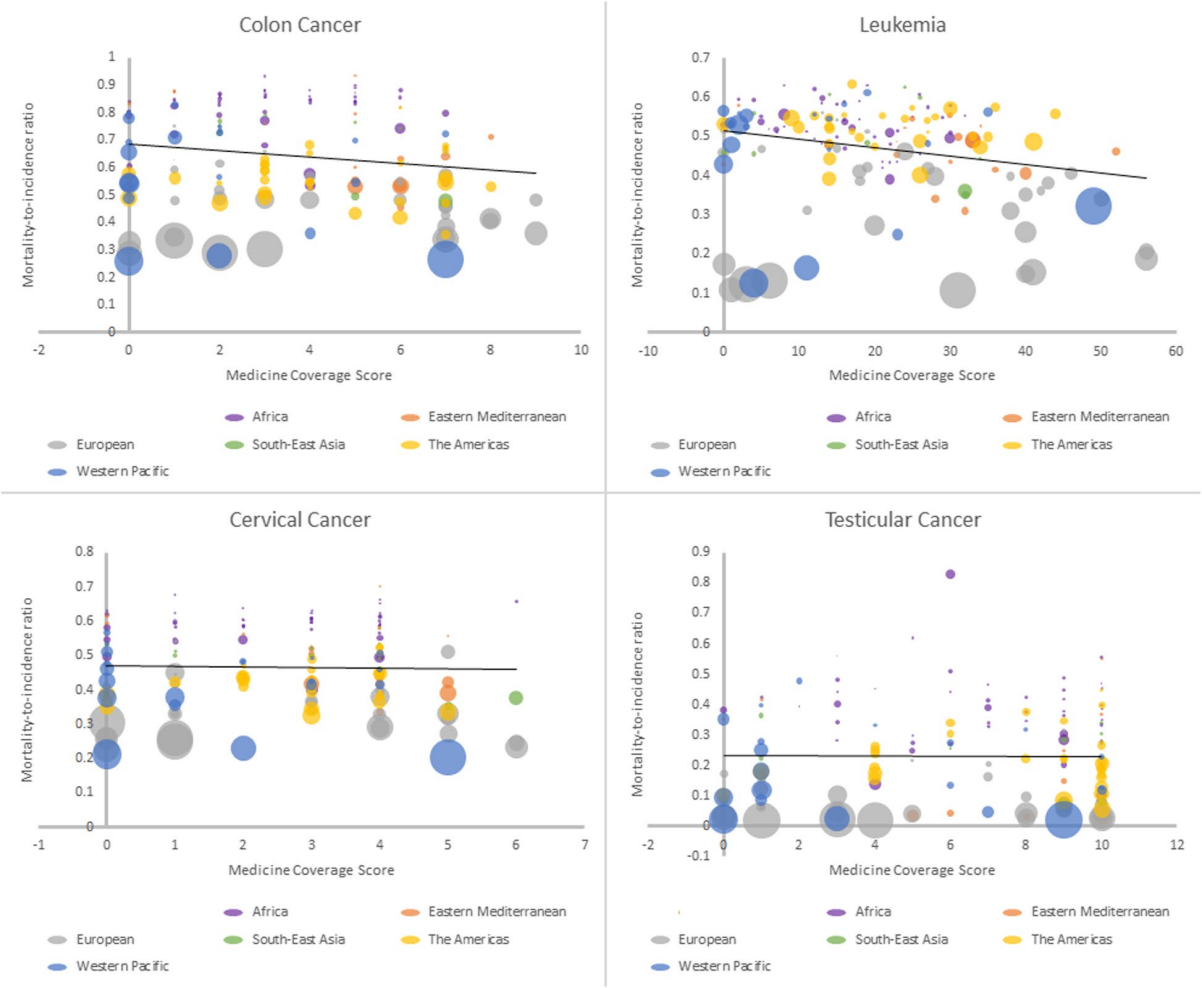
Relationship between medicine coverage score and mortality-to-incidence ratio for colon cancer, leukemia, cervical cancer, and testicular cancer, with the size of the bubbles representing healthcare expenditure

The relationship between variables by regions, with the size of the bubbles representing healthcare expenditure for all eight cancers is shown in Figs. 1 and 2. Figure 1 depicts a decrease in MIR as coverage score increases for non-melanoma skin cancer, uterine cancer, breast cancer, and Hodgkin lymphoma. Figure 2 depicts a decrease in MIR as coverage score increases for colon cancer and leukemia, but not for cervical cancer or testicular cancer.

We present results of two of our regression models in Table 2. Briefly, there were associations between medicine listing and mortality outcomes for six of the eight examined cancers (non-melanoma skin cancer (*p* = 0.001), uterine cancer (*p* = 0.006), breast cancer (*p* = 0.001), Hodgkin lymphoma (*p* = 0.021), colon cancer (*p* = 0.006), and leukemia (*p* = 0.002) when adjusting for healthcare expenditure and population size. For each of the eight cancer causes, the relationship between listing medicines and cancer outcomes are detailed below.

**Table 2:** Regression results

	MIR with coverage score, adjusted for healthcare expenditure and population size					MIR with healthcare expenditure				
	Beta-Coefficient	***P***-value	Lower 95% CI	Upper 95% CI	Adjusted *R*^2^ of model	Beta-Coefficient	***P***-value	Lower 95% CI	Upper 95% CI	Adjusted *R*^2^ of model
**NM Skin**	−0.0229575	0.001	−0.0360089	−0.0099062	0.2169	−0.000104	< 0.0001	−0.0001404	−0.0000677	0.1647
**Uterine**	−0.019615	0.006	−0.0334501	−0.0057798	0.2989	−0.00005	< 0.0001	−0.0000629	−0.0000372	0.2701
**Breast**	−0.0032446	0.001	−0.0051592	0.0013301	0.3983	−0.0000839	< 0.0001	−0.0001015	−0.0000663	0.3591
**Hodgkin**	−0.0070121	0.021	−0.129642	0.0010599	0.4065	−0.0000839	< 0.0001	−0.0001015	−0.0000663	0.3591
**Colon**	−0.0117881	0.006	−0.0200667	−0.0035094	0.4773	−0.0000958	< 0.0001	−0.0001124	−0.0000793	0.4528
**Leukemia**	−0.001513	0.002	−0.0024832	−0.0005429	0.5387	−0.000678	< 0.0001	−0.0000782	−0.0000574	0.5123
**Cervical**	−0.0051497	0.214	−0.0133043	0.0030049	0.4319	−0.0000607	< 0.0001	−0.0000716	−0.0000498	0.4334
**Testicular**	−0.0044925	0.145	−0.0105539	0.0015688	0.2674	−0.0000648	< 0.0001	−0.0000818	−0.0000477	0.2604

### Non-melanoma skin cancer

The unadjusted model demonstrated that listing the medicines for non-melanoma skin cancer was negatively associated with the mortality to incidence ratio (*b* = −0.016 indicating that for each additional cancer medicine listed the MIR decreased by 0.016, *p* = 0.024). The proportion of variance in the MIR explained by a country listing non-melanoma skin cancer medicines was 2.6% (adjusted *R*^2^ = 0.026). After adjusting for healthcare expenditure and population size, we found a negative association between listing non-melanoma skin cancer medicines and MIR (*b* = −0.023, *p* = 0.001). In this adjusted model, the number of medicines listed on NEMLs along with healthcare expenditure and population size accounted for 21.7% of the differences in non-melanoma cancer MIR (adjusted *R*^2^ = 0.217).

### Uterine cancer

In both the unadjusted and adjusted models, listing uterine cancer medicines was negatively associated with countries’ MIRs (*b* = −0.027, *p* = 0.001; and *b* = −0.020, *p* = 0.006, respectively). The number of medicines explained 6.1% of the variance in the MIR (adjusted *R*^2^ = 0.061). The number of medicines, healthcare expenditure and population size explained 29.9% of the variance in the MIR (adjusted *R*^2^ = 0.299).

### Breast cancer

In the unadjusted model, listing breast cancer medicines was negatively associated with countries’ breast cancer mortality-to-incidence ratios (*b* = −0.004, *p* = 0.001). The number of listed medicines explained 5.7% of the variance in the MIR (adjusted *R*^2^ = 0.057). The adjusted model also demonstrated a significant association between listing breast cancer medicines and the countries’ breast cancer MIRs (*b* = −0.003, *p* = 0.001). The adjusted model accounted for 39.8% of the variance in the MIR (adjusted *R*^2^ = 0.398).

### Hodgkin lymphoma

For Hodgkin lymphoma, listing cancer medicines was not significantly associated with the countries’ MIRs in the unadjusted model (*b* = −0.004, *p* = 0.356). The unadjusted regression model showed that listing Hodgkin lymphoma medicines accounted for little of the variance in MIR (adjusted *R*^2^ = −0.0009). In adjusted model, listing Hodgkin lymphoma medicines was negatively associated with MIR (*b* = −0.007, *p* = 0.021). The adjusted model accounted for 40.6% of the variance in the MIR (adjusted *R*^2^ = 0.406).

### Colon cancer

The unadjusted model demonstrated that listing the medicines for colon cancer was negatively associated with countries’ colon cancer MIRs (*b* = −0.012, *p* = 0.039). Listing colon cancer medicines accounted for 2.1% of the variance in countries MIRs (adjusted *R*^2^ = 0.021). The adjusted model showed a significant association between listing colon cancer medicines and countries’ colon cancer MIRs (*b* = −0.012, *p* = 0.006). The adjusted model accounted for 47.7% of the variance in countries’ MIRs (adjusted *R*^2^ = 0.477).

### Leukemia

The unadjusted model demonstrated that listing the medicines for leukemia was negatively associated with countries’ colon cancer MIRs (*b* = −0.002, *p* = 0.003). For the unadjusted model, 5.1% of the variance in countries’ leukemia MIRs were explained by listing leukemia medicines (adjusted *R*^2^ = 0.051). In the adjusted model, there was also a significant association between listing leukemia medicines and countries’ leukemia MIRs (*b* = −0.002, *p* = 0.002). For the adjusted model, 53.9% of the variance in countries’ leukemia MIRs were explained by listing leukemia medicines, healthcare expenditure, and population size (adjusted *R*^2^ = 0.539).

### Cervical cancer

For cervical cancer, listing cancer medicines was not significantly associated with cervical cancer MIR in both the unadjusted (*b* = −0.001, *p* = 0.785) and adjusted (*b* = −0.005, *p* = 0.214) models. The unadjusted regression model showed that listing medicines for cervical cancer accounted for little of the variance in MIR (adjusted *R*^2^ = −0.006), and the adjusted model showed that listing medicines for cervical cancer, as well as healthcare expenditure and population size accounted for 43.2% of the variance in MIR (adjusted *R*^2^ = 0.432).

### Testicular cancer

For testicular cancer, listing testicular cancer medicines was not significantly associated with testicular cancer MIR in both the unadjusted (*b* = −0.003, *p* = 0.930) and adjusted (*b* = −0.004, *p* = 0.145) models. The unadjusted regression model showed that listing medicines for testicular cancer accounted for little of the variance in MIR (adjusted *R*^2^ = −0.006), and the adjusted model accounted for 26.7% of the variance in MIR (adjusted *R*^2^ = 0.267).

## Discussion

Listing essential medicines for NM skin cancer, uterine cancer, breast cancer, Hodgkin lymphoma, colon cancer, and leukemia was associated with better disease-specific avoidable mortality when controlling for healthcare expenditure and population size, but this relationship was not present for cervical cancer and testicular cancer. The most commonly added medicines were docetaxel, anastrozole, capecitabine, oxaliplatin, and paclitaxel, and some new cancer medicines were listed that were not listed in previously in any NEML.

Our findings align with our previous study that found only a weak association between listing cancer treatments and relevant cancer outcomes [13]. These results could be explained by the listed medicines not being available or accessible, by aspects of cancer care outside of medicine access, or by limits of the benefits of cancer treatments. While including cancer medicines on NEMLs is necessary to establish government priorities and standards, these priorities do not always translate into access for patients. Essential cancer medicines may not always be accessible to those who need them, and there are barriers to availability and affordable access to cancer medicines. A 2017 study of 63 countries found that that many of the cancer medicines listed in the WHO EML were either not available at all, or only available at full cost, particularly in low-income and low-middle-income countries [30]. Several countries have reported low availability of cancer medicines as well as lack of affordability [31–41]. Some high-income countries also face lack of availability and affordability of cancer medicines [30, 39, 42–44]. Poor cancer medicine availability can result in treatment changes and delays that may impact medicine efficacy and toxicity [44–47]. Some patients may forgo cancer medicines altogether [31]. Furthermore, NEMLs do not always align with other national policies and systems, such as procurement and distribution, and drug reimbursement and coverage policies [48, 49].

Cancer medicines represent only one subset of cancer care, and other aspects of the cancer care continuum contribute to cancer outcomes. Improved prevention, screening, and diagnosis programs have contributed to greater reductions in cancer mortality in high-income countries compared to lower-income countries [50, 51]. Other treatment modalities such as radiation therapy also play a role in cancer outcomes [51, 52]. Many countries lack sufficient radiotherapy infrastructure, due to the high upfront costs of obtaining the machines [51, 53].

In particular, the lack of an association seen with testicular cancer may be explained by the greater role of surgical interventions in testicular cancer treatment [54]. For cervical cancer, which also did not show an association with cancer medicine listing, screening may play a role in mortality [55]. Notably, for all eight cancers, there is a large difference between the variance in the unadjusted and adjusted models, indicating that adjusting for population size and healthcare expenditure has an important influence. This aligns with findings that demonstrate associations between healthcare expenditures and cancer mortality [56, 57].

Our findings indicate a weak relationship between listing cancer treatments and cancer outcomes; most NEMLs list established cancer medicines, and this raises questions about whether listing newer medicines would greatly reduce cancer mortality. Evidence supporting the treatment benefits of novel cancer medicines is not always robust, and some studies of newer cancer treatments have a high risk of bias [58–60]. From 2018 to 2024, 85% of cancer medicine launches had annual costs above $100,000 USD, and 55% of new cancer medicines launches had annual costs above $200,000 USD [61]. However, it has been reported that there is no meaningful association between cancer medicines prices and the magnitude of benefit [62]. When considering adding newer cancer treatments to NEMLs, treatment benefits, as well as financial costs and affordability should be taken into account.

### Strengths and limitations

A major strength of this study is that we included a large number of countries; we identified 158 countries with NEMLs and obtained disease-specific MIRs, healthcare expenditure, and population size data for all 158 countries. However, NEMLs for other countries may exist but may not have been identified in our searches. Furthermore, we used a robust search method to identify clinically appropriate medicines for each of the eight cancers, using WHO guidelines, MEDI-HPS and MIA databases, and the WHO 2023 EML. Finally, we used MIR as our outcome variable, which is often used as a measure of cancer outcomes since it is based on mortality and incidence data that is readily available in several countries. However, this measure relies on mortality and incidence being accurately recorded and may be affected by non-treatment related factors such as screening and diagnosis, and other cancer treatments such as radiation and surgery may affect cancer outcomes. Furthermore, there are variations in how countries use NEMLs, and many countries face barriers in implementing NEMLs, which can impact medicine access [48]. There is limited available information about how NEMLs are utilized and implemented in different countries, which can impact the robustness of our findings.

### Implications for policy and research

Our findings indicate that simply listing cancer medicines on NEMLs play a small role in reducing cancer mortality. Policymakers should consider the importance of improving equitable access to cancer treatments, which requires investments in healthcare capacity and resourcing at local, national, and international levels. As well, our findings highlight the need to address cancer care through several modalities, including screening and diagnosis, indicating the need for continued investments in preventative care programs that reach all populations, including people who experience disadvantages. Further research could include country-specific analyses to capture the effects of listing cancer medicines while accounting for how the NEML is utilized, and how medicines are made available to the population. Future research could also examine the cost of cancer medicines in relation to their inclusion on NEMLs and effects on cancer mortality.

## Conclusion

There was an association between listing cancer medicines on NEMLs and cancer mortality. Further research is required to explore how cancer mortality may be impacted by other cancer interventions (such as screening, diagnosis, surgery, and radiation), and policies to improve equitable access to cancer care.

## Supplementary Information

Supplementary Material 1.

## Data Availability

The datasets used and analysed during the current study are available from the corresponding author on reasonable request.

## Abbreviations

EML: Essential medicines list
GEM: Global essential medicines
NEML: National essential medicines list
WHO: World Health Organization
