# Supplementary material for: Changes in essential cancer medicines and association with cancer outcomes: an observational study of 158 countries
Source: BMC Cancer. 2024 Dec 18;24:1526. doi: 10.1186/s12885-024-13247-w (PMC11656608; doi:10.1186/s12885-024-13247-w)
Supplement: Supplementary file 1 — Supplementary Material 1. [file 12885_2024_13247_MOESM1_ESM.docx]

Appendix. Medicines indicated for non-melanoma skin cancer, uterine cancer, breast cancer, Hodgkin lymphoma, colon cancer, leukemia, cervical cancer, and testicular cancer

| Cancer | Medicine Name | ATC Code |
| --- | --- | --- |
| Non-melanoma skin cancer | podophyllotoxin | D06BB04 |
|  | imiquimod | D06BB10 |
|  | ifosfamide | L01AA06 |
|  | fluorouracil | L01BC02 |
|  | capecitabine | L01BC06 |
|  | vinblastine | L01CA01 |
|  | vincristine | L01CA02 |
|  | paclitaxel | L01CD01 |
|  | doxorubicin (adriamycin) | L01DB01 |
|  | daunorubicin | L01DB02 |
|  | bleomycin | L01DC01 |
|  | avelumab | L01FF04 |
|  | vismodegib | L01XJ01 |
|  | sonidegib | L01XJ02 |
| Uterine cancer | megestrol | G03AC05 |
|  | medroxyprogesterone | G03AC06 |
|  | cetrorelix | H01CC02 |
|  | paclitaxel | L01CD01 |
|  | topotecan | L01CE01 |
|  | amifostine | V03AF05 |
| Breast cancer | megestrol | G03AC05 |
|  | medroxyprogesterone | G03AC06 |
|  | cyclophosphamide | L01AA01 |
|  | chlorambucil | L01AA02 |
|  | melphalan | L01AA03 |
|  | chlormethine (mechlorethamine, mustine) | L01AA05 |
|  | ifosfamide | L01AA06 |
|  | prednimustine | L01AA08 |
|  | bendamustine | L01AA09 |
|  | thiotepa | L01AC01 |
|  | fluorouracil | L01BC02 |
|  | tegafur and uracil | L01BC03 |
|  | gemcitabine | L01BC05 |
|  | capecitabine | L01BC06 |
|  | azacitidine | L01BC07 |
|  | decitabine | L01BC08 |
|  | vinblastine | L01CA01 |
|  | vinorelbine | L01CA04 |
|  | paclitaxel | L01CD01 |
|  | docetaxel | L01CD02 |
|  | irinotecan | L01CE02 |
|  | doxorubicin (adriamycin) | L01DB01 |
|  | daunorubicin | L01DB02 |
|  | epirubicin | L01DB03 |
|  | idarubicin | L01DB06 |
|  | mitoxantrone | L01DB07 |
|  | ixabepilone | L01DC04 |
|  | palbociclib | L01EF01 |
|  | abemaciclib | L01EF03 |
|  | temsirolimus | L01EG01 |
|  | everolimus | L01EG02 |
|  | lapatinib | L01EH01 |
|  | neratinib | L01EH02 |
|  | trastuzumab | L01FD01 |
|  | pertuzumab | L01FD02 |
|  | atezolizumab | L01FF05 |
|  | bevacizumab | L01FG01 |
|  | cisplatin | L01XA01 |
|  | carboplatin | L01XA02 |
|  | eribulin | L01XX41 |
|  | buserelin | L02AE01 |
|  | leuprorelin (leuprolide) | L02AE02 |
|  | goserelin | L02AE03 |
|  | triptorelin | L02AE04 |
|  | histrelin | L02AE05 |
|  | tamoxifen | L02BA01 |
|  | toremifene | L02BA02 |
|  | fulvestrant | L02BA03 |
|  | aminoglutethimide | L02BG01 |
|  | anastrozole | L02BG03 |
|  | letrozole | L02BG04 |
|  | exemestane | L02BG06 |
|  | filgrastim | L03AA02 |
|  | methotrexate | L04AX03 |
|  | dexrazoxane | V03AF02 |
| Hodgkin's lymphoma | cyclophosphamide | L01AA01 |
|  | chlorambucil | L01AA02 |
|  | melphalan | L01AA03 |
|  | chlormethine (mechlorethamine, mustine) | L01AA05 |
|  | ifosfamide | L01AA06 |
|  | prednimustine | L01AA08 |
|  | bendamustine | L01AA09 |
|  | carmustine | L01AD01 |
|  | lomustine | L01AD02 |
|  | semustine | L01AD03 |
|  | streptozocin | L01AD04 |
|  | fotemustine | L01AD05 |
|  | uramustine | L01AD08 |
|  | dacarbazine | L01AX04 |
|  | vinblastine | L01CA01 |
|  | vincristine | L01CA02 |
|  | vindesine | L01CA03 |
|  | vinorelbine | L01CA04 |
|  | vinflunine | L01CA05 |
|  | etoposide | L01CB01 |
|  | doxorubicin (adriamycin) | L01DB01 |
|  | bleomycin | L01DC01 |
|  | pembrolizumab | L01FF02 |
|  | brentuximab vedotin | L01FX05 |
|  | cisplatin | L01XA01 |
|  | procarbazine | L01XB01 |
| Colon cancer | raltitrexed | L01BA03 |
|  | fluorouracil | L01BC02 |
|  | capecitabine | L01BC06 |
|  | irinotecan | L01CE02 |
|  | regorafenib | L01EX05 |
|  | cetuximab | L01FE01 |
|  | bevacizumab | L01FG01 |
|  | oxaliplatin | L01XA03 |
|  | calcium folinate (folinic acid, leucovorin) | V03AF |
|  | calcium levoflinate (levofolinic acid) | V03AF04 |
| Leukemia | tretinoin (transretinoic acid) | D10AD01 |
|  | isotretinoin | D10AD04 |
|  | ruxolitinib | D11AH09 |
|  | cyclophosphamide | L01AA01 |
|  | chlorambucil | L01AA02 |
|  | melphalan | L01AA03 |
|  | chlormethine (mechlorethamine, mustine) | L01AA05 |
|  | ifosfamide | L01AA06 |
|  | prednimustine | L01AA08 |
|  | bendamustine | L01AA09 |
|  | busulfan | L01AB01 |
|  | streptozocin | L01AD04 |
|  | dacarbazine | L01AX04 |
|  | mercaptopurine | L01BB02 |
|  | tioguanine | L01BB03 |
|  | cladribine | L01BB04 |
|  | fludarabine | L01BB05 |
|  | clofarabine | L01BB06 |
|  | nelarabine | L01BB07 |
|  | cytarabine | L01BC01 |
|  | fluorouracil | L01BC02 |
|  | tegafur and uracil | L01BC03 |
|  | gemcitabine | L01BC05 |
|  | capecitabine | L01BC06 |
|  | azacitidine | L01BC07 |
|  | decitabine | L01BC08 |
|  | vincristine | L01CA02 |
|  | vindesine | L01CA03 |
|  | etoposide | L01CB01 |
|  | teniposide | L01CB02 |
|  | topotecan | L01CE01 |
|  | irinotecan | L01CE02 |
|  | dactinomycin | L01DA01 |
|  | doxorubicin (adriamycin) | L01DB01 |
|  | daunorubicin | L01DB02 |
|  | epirubicin | L01DB03 |
|  | idarubicin | L01DB06 |
|  | mitoxantrone | L01DB07 |
|  | pixantrone | L01DB11 |
|  | imatinib | L01EA01 |
|  | dasatinib | L01EA02 |
|  | nilotinib | L01EA03 |
|  | bosutinib | L01EA04 |
|  | ponatinib | L01EA05 |
|  | ibrutinib | L01EL01 |
|  | idelalisib | L01EM01 |
|  | midostaurin | L01EX10 |
|  | rituximab | L01FA01 |
|  | ofatumumab | L01FA02 |
|  | obinutuzumab | L01FA03 |
|  | inotuzumab ozogamicin | L01FB01 |
|  | gemtuzumab ozogamicin | L01FX02 |
|  | blinatumomab | L01FX07 |
|  | cisplatin | L01XA01 |
|  | amsacrine | L01XX01 |
|  | asparaginase (crisantaspase recombinant) | L01XX02 |
|  | hydroxycarbamide (hydroxyurea) | L01XX05 |
|  | pentostatin (deoxycoformycin) | L01XX08 |
|  | mitotane | L01XX23 |
|  | pegaspargase | L01XX24 |
|  | arsenic trioxide | L01XX27 |
|  | anagrelide | L01XX35 |
|  | venetoclax | L01XX52 |
|  | interferon gamma | L03AB03 |
|  | interferon alfa-2a | L03AB04 |
|  | interferon alfa-2b | L03AB05 |
|  | interferon beta-1b | L03AB08 |
|  | peginterferon alfa-2b | L03AB10 |
|  | peginterferon alfa-2a | L03AB11 |
|  | alemtuzumab | L04AA34 |
|  | methotrexate | L04AX03 |
|  | lenalidomide | L04AX04 |
|  | rasburicase | V03AF07 |
| Cervical cancer | papillomavirus vaccines | J07BM |
|  | ifosfamide | L01AA06 |
|  | paclitaxel | L01CD01 |
|  | topotecan | L01CE01 |
|  | pembrolizumab | L01FF02 |
|  | cisplatin | L01XA01 |
| Testicular cancer | chlorambucil | L01AA02 |
|  | ifosfamide | L01AA06 |
|  | vinblastine | L01CA01 |
|  | etoposide | L01CB01 |
|  | dactinomycin | L01DA01 |
|  | bleomycin | L01DC01 |
|  | cisplatin | L01XA01 |
|  | carboplatin | L01XA02 |
|  | methotrexate | L04AX03 |
|  | mesna | R05CB05 |
